# Reality of evidence-based practice in palliative care

**DOI:** 10.7497/j.issn.2095-3941.2015.0041

**Published:** 2015-09

**Authors:** Claire Visser, Gina Hadley, Bee Wee

**Affiliations:** ^1^Harris Manchester College, University of Oxford, Oxford OX3 9DU, UK; ^2^Sir Michael Sobell House, Oxford University Hospitals NHS Trust, Oxford OX3 9DU, UK

**Keywords:** Palliative care, evidence-based medicine (EBM), multimorbidity, clinical meaningfulness

## Abstract

There has been a paradigm shift in medicine away from tradition, anecdote and theoretical reasoning from the basic sciences towards evidence-based medicine (EBM). In palliative care however, statistically significant benefits may be marginal and may not be related to clinical meaningfulness. The typical treatment *vs*. placebo comparison necessitated by ‘gold standard’ randomised controlled trials (RCTs) is not necessarily applicable. The complex multimorbidity of end of life care involves considerations of the patient’s physical, psychological, social and spiritual needs. In addition, the field of palliative care covers a heterogeneous group of chronic and incurable diseases no longer limited to cancer. Adequate sample sizes can be difficult to achieve, reducing the power of studies and high attrition rates can result in inadequate follow up periods. This review uses examples of the management of cancer-related fatigue and death rattle (noisy breathing) to demonstrate the current state of EBM in palliative care. The future of EBM in palliative care needs to be as diverse as the patients who ultimately derive benefit. Non-RCT methodologies of equivalent quality, validity and size conducted by collaborative research networks using a ‘mixed methods approach’ are likely to pose the correct clinical questions and derive evidence-based yet clinically relevant outcomes.

## Introduction

Evidence-based medicine (EBM) is “the conscientious, explicit, and judicious use of current best evidence in making decisions about the care of individual patients”^1^. EBM has rapidly developed over the past 20 years. A recent quantification of research output reported that approximately 75 trials and 11 systematic reviews are published daily by the medical research community^2^, and this volume continues to increase each year. The central role of this ever-expanding bank of evidence in clinical decision-making has changed practice irrevocably. Initiation of a treatment on the basis that our colleagues deem it effective or we ourselves believe that such treatment will work is no longer acceptable. We must carefully scrutinize the available evidence, appraise its quality, and estimate its applicability to a specific patient before discussing available treatment options.

Despite inherent challenges, the EBM approach has resulted in tangible and counter-intuitive improvements in patient care by rationalizing clinical scenarios, such as anti-arrhythmic drug use^3^, venous thromboembolism prevention^4^, and acute asthma management^5^. EBM is regarded as a non-negotiable part of modern clinical practice. However, to conclude that EBM has equally permeated all medical specialties would be incorrect. Professor David Sackett has commented on the development of EBM in the UK, quoting the American author William Gibson: “*The future is already here—it’s just not very evenly distributed*”^6^. Indeed, palliative care^7^ and other clinical fields, such as psychiatry^8^ have lagged significantly behind their counterparts in their development and use of EBM, because of the incompatibility of the fundamental assumptions and methodologies of EBM with the reality of their clinical contexts.

Palliative care is defined by the World Health Organization as “*an approach that improves the quality of life of patients and their families facing the problem associated with life-threatening illness, through the prevention and relief of suffering by means of early identification and impeccable assessment and treatment of pain and other problems, physical, psychosocial and spiritual*”^9^. Whilst the need for palliative care is often associated with a diagnosis of cancer, the increasing prevalence of other chronic and incurable diseases^10^ has expanded the field into one, attempting to satisfy the needs of patients with a wide range of diseases. This heterogeneity of patient diagnosis, symptomatology, and disease stage presents a unique challenge for those who attempt to conduct research in palliative care.

This review discusses the challenges of conducting high-quality research in palliative care and the limitations of EBM when applied to a field that does not fit the framework of the traditional EBM approach. This review also highlights the role of collaborative groups in palliative care in strengthening research infrastructure and improving trial quality. Moreover, this paper discusses the functions of well-designed observational studies in augmenting the inadequacy of high-quality evidence in palliative care. This review further discusses the development of the evidence base in two specific areas of palliative care research, that is, cancer-related fatigue management and death rattle (noisy breathing) at the end of life, to highlight the achievements and the remaining gaps in the palliative care evidence base.

## High-quality evidence is needed in palliative care

The palliative care literature has expanded, with the number of clinical trials in palliative care increasing from 0.2% to 0.8% between 1970 and 2005^11^. However, as a percentage of the overall palliative care literature, published, clinical trials have remained only a small portion of the research output in the field^11^. A comprehensive analysis of the publication patterns of palliative care research has revealed an insufficiency of high-quality controlled trials. Hui *et al*.^12^ compared the palliative care literature published in the first 6 months of 2004 with that in the first 6 months of 2009 by using a systematic search and descriptive analysis of the 1,213 studies involved. The studies were categorized by variables such as quality and design. Randomised controlled trials (RCTs) comprised only 6% of the original studies identified. The proportion of original studies also increased between 2004 and 2009. Nevertheless, the proportion of analytical studies, that is, those with interventions, either cohort studies or RCTs remained static over time. The authors concluded the presence of “*significant deficiencies in the quantity, design, and scope of the palliative care literature*”. This lack of quality primary literature has affected the utility of more rigorous methods of information gathering, such as systematic reviews. Wee *et al*.^13^ reviewed the primary studies for 25 published Cochrane Systematic Reviews on palliative care interventions and assessed the quality of the interventions performed. Only eight of the reviews contained more than 1,000 participants, with the majority examining data obtained from less than 500 study patients. Most of the primary studies reviewed were methodologically flawed. Even those trials considered to be of higher quality were inadequately powered. Despite the successful review process, numerous issues persisted and as a result, all but two systematic reviews did not provide strong evidence in favor of an intervention. The authors concluded that “*Cochrane reviews in palliative care are well performed, but fail to provide good evidence to guide clinical practice because the primary studies are few in number, small, clinically heterogeneous, and of poor quality and external validity*”. As an extension of a previous systematic assessment of the palliative care literature, Hui *et al*.^14^ specifically examined RCTs published in the first 6 months of 2004 and 2009. They assessed a total of 44 RCTs and found that aside from the small sample sizes, the majority of RCTs did not properly report trial methodology, including randomization and allocation concealment, sample size calculation, and blinding. The authors concluded that the overall quality of the methodology and its reporting was “poor”; echoing well-documented concerns about the availability, quality, and quantity of the palliative care literature^7^^,^^12^^,^^15^^-^^18^.

## Palliative care does not fit the EBM framework

RCTs are considered the “gold standard” for evaluating the efficacy of an intervention because of their ability to minimize bias^19^^,^^20^. The validity of such trials is judged based on universal and objective methodological criteria^21^^-^^23^. Conducting high-quality research in the palliative care setting remains challenging. First, high-quality studies must be adequately powered. This feature involves the recruitment of a large sample of consenting patients. Stone *et al*.^24^ documented the experience of recruiting participants for a large observational study to evaluate a prognostic instrument for palliative care. The authors described a low-percentage recruitment rate, with only 8% of the screened patients eventually enrolled in the trial. They identified specific barriers to recruitment, which included a lack of patient “accessibility”. This concept was subjectively defined by the researchers and comprised scenarios in which patients either deteriorated rapidly or died before being enrolled; were too cognitively impaired to consent, or were prevented from enrolling because of “gatekeeping”^25^^,^^26^ by relatives and clinical staff. This study exemplifies some of the commonly cited issues that prevent many studies in palliative care from achieving adequate sample sizes^27^. Other concerns about recruitment include the putative belief of patients and their guardians that enrolling in a trial would add burden to an already physically and emotionally challenging time. Whilst these views are to an extent borne out in a recent systematic review of qualitative work, the authors note that there is considerable overall willingness to participate in palliative care research, despite mention of some specific deterrents^28^. Nevertheless, RCTs were generally met with the most suspicion in the studies reviewed, with only 40% of patients indicating that they would be happy to participate in a RCT. The authors suggested that recruitment is most likely to be successful in simple, non-invasive, and low-burden studies, in which the experimental design is well explained.

Second, high-quality trials must have a follow-up period of appropriate length and completeness. This criterion is particularly vital to assess the validity of RCTs, in which an intention to treat the analysis results relies upon the completeness of the data set. Hui *et al*.^29^ reviewed 18 prospective clinical trials, of which 15 were randomized and conducted in a single palliative care center between 1999 and 2011. The median attrition rate to reach the end of the trial was 44%. The vast majority of this attrition was attributed to patient withdrawal, with the most common withdrawal reason cited as an increase in symptom burden. Whilst the focus on a single site could make the study difficult to generalize, very few site or trial-related factors seemed to influence the attrition rate. Loss to follow up, protocol violations, and safety concerns minimally contributed to the overall attrition rate. Hence, patient factors played the largest role. This finding suggests that if other palliative patient populations have a similar symptom burden, which is likely, then attrition caused by symptomatic deterioration could be high in numerous studies in the palliative care setting. Indeed, some reports suggest that palliative care patients may have a median of 11 different symptoms^30^.

For a study to have validity there should be a clear link between interventions and outcomes. This phenomenon can be achieved by selecting a homogenous population, utilizing a single well-defined intervention, and analyzing outcomes with objective and clinically relevant end-points. This paradigm presents three further problems for the field of palliative care. First, the common practice of implementing packages of palliative treatments for multiple symptoms causes difficultly in controlling for the effects of interventions, additional to the effect under study. The holistic care of palliative care patients involves and is not limited to addressing their physical, psychological, social, and spiritual needs. Second, the heterogeneity of the palliative care population requires a balance between trial validity and trial generalizability; achieving this balance is a complex task^31^. Lastly, the traditional end points used in RCTs, such as death and disability, are largely inappropriate for the palliative population. The goal of palliative care research should be to improve quality of life. This amorphous concept has many components, which lend themselves poorly to operationalization. The psychological and spiritual aspects of palliative care are the most difficult to study by using quantitative methods; by contrast, research groups in other areas, such as pain research, have reported reasonable success with validated rating scales^32^^-^^35^. However well-characterised, the study of such variables undoubtedly benefits from blinding to reduce bias within inherently subjective assessments^36^. This process is difficult to achieve in many palliative care research settings. Indeed the ‘validity’ of statistical significance in the context of palliative care is debatable, as it may not correlate at all with clinical meaningfulness.

Numerous other factors contribute to the success of a particular field in the development of a sound evidence base. These factors include, but are not limited to, ethical complexity^37^^-^^40^, under-developed research infrastructure or funding^41^, and difficulty in accessing existing research data^17^. These issues are particularly relevant to palliative care but are beyond the scope of the present review and will not be further discussed.

## Improving palliative care research

Given that RCTs are difficult to conduct in the palliative care setting and, at times, inappropriate for practical and ethical reasons, researchers in the field have looked to alternative research methodologies, including observational studies^42^. These studies can yield equivalent results to RCTs if they are of high quality, have a large sample size, and are methodologically valid^43^^,^^44^. As such, received wisdom regarding the “hierarchy of evidence” has been challenged^45^. Hadley *et al*.^46^ attempted to identify observational studies in the field of palliative care that satisfied the criteria of quality, validity, and size to establish whether or not they could be a realistic alternative to RCTs. This review identified 340 studies, 91% of which were not obtained from easily accessible sources, such as PubMed, but from communication with experts in the field. The resulting studies were few in number, heterogeneous in methodology and patient group, and small (only one quarter contained more than 200 subjects). Therefore, the authors concluded that there is a “*deficiency of large, good quality observational studies of particular interventions in particular patients with particular and defined outcomes*”.

A recent Cochrane systematic review of sedation at the end of life care^47^ selected 14 trials, of which none were RCTs and only four were prospective observational studies. Three quarters of the studies had sample sizes of less than 600, and only one study attempted to perform group matching. The authors concluded that the overall quality of the included studies was low. Meanwhile, the analysis of the scope and quality of the palliative care literature by Hui *et al*.^12^ revealed that only 59% of studies in the period assessed were prospective in nature, which is a vital feature of a high-quality observational study. Therefore, it seems unlikely that observational studies of sufficient quality to rival RCTs have become abundant since publication of the literature review of Hadley *et al*.^46^.

Despite the deficiency of good quality observational studies, a recent large-scale, multi-centered, open-label prospective registry study was conducted in Italy^48^^,^^49^. In a period of 1 year, the team recruited 1,801 individual cases from over 100 centers, which provided a large and data-rich resource on the epidemiology and treatment of cancer breakthrough pain. This study demonstrated that with a collaborative research approach, sufficiently large sample sizes can be obtained to satisfy the criteria of high-quality observational studies. Other efforts to expand palliative care research have focused on this type of collaborative organization, which is derived from the design of oncology trials which are characteristically multi-centered, collaborative, and of high quality^50^. The establishment of palliative care collaborative groups has been widespread, with the US and Europe^16^^,^^51^^,^^52^ both uniting researchers in the field into cooperative groups in the hope that large-scale high quality and multi-site randomised studies would be possible. Three such trials have recently assessed the effectiveness of modafinil, which is a selective central nervous system stimulant, for the treatment of cancer-related fatigue, and have demonstrated the valuable contribution to the palliative care literature that can be made by well-controlled research trials^53^^-^^55^. The most recent study was a multi-site, randomized, double-blinded, placebo-controlled trial, in which 206 patients with non-small cell lung cancer were randomized at a 1:1 ratio to either modafinil or placebo treatment for 28 days^53^. The primary outcome measure was a change in the FACIT-Fatigue score, a validated 13-item measure of fatigue development in oncology patients^56^. The trial demonstrated a clinically meaningful improvement in the FACIT-Fatigue score for patients in both the modafinil and placebo arms, concluding that there was no significant difference in the effectiveness of modafinil for fatigue compared with placebo. Secondary outcome measures, including measures of daytime sleepiness, depression, quality of life, and toxicity as well as adverse event rates, were also equivalent between the groups. This study was well designed and conducted, with features of high methodological quality, such as stratification by clinically significant variables, including performance status, and a rigorous randomization protocol^21^. This study was preceded by a pilot study from which sample-size estimates were derived; as a result, the study was sufficiently powered^57^. Two previous trials also failed to determine a statistically significant overall difference between modafinil and placebo treatments, although one trial^54^ found that a subgroup with severe baseline fatigue benefitted from modafinil as assessed by the results of the Brief Fatigue Inventory. The smallest trial^55^ found no significant difference in primary or subgroup analyses. The results of these studies are significant because they provide important data on the null effect of a treatment which is associated with potential side effects, including headache, anxiety, nausea, and dizziness. Whilst initial assessments of the neurochemical effects of modafinil have suggested that its tolerability is reasonable, and that it is preferable to traditional stimulants, such as amphetamines^58^. Arguably the term “tolerability” has different meanings in different clinical contexts. In the palliative setting, in which symptom management is a core aim, the addition of symptoms to an already burdened patient should not be lightly dismissed. By highlighting the limited effectiveness of this drug treatment relative to placebo, these studies have significantly contributed by preventing over-treatment with modafinil, thereby limiting unnecessary side effects among patients. Such a finding demonstrates the value of null results in methodologically robust studies. Indeed, counter-intuitive results are arguably the most valuable, as they are by definition findings that would not have become evident unless subjected to empirical scrutiny.

## The leak between evidence and practice: treatment of death rattle

**Figure 1:**
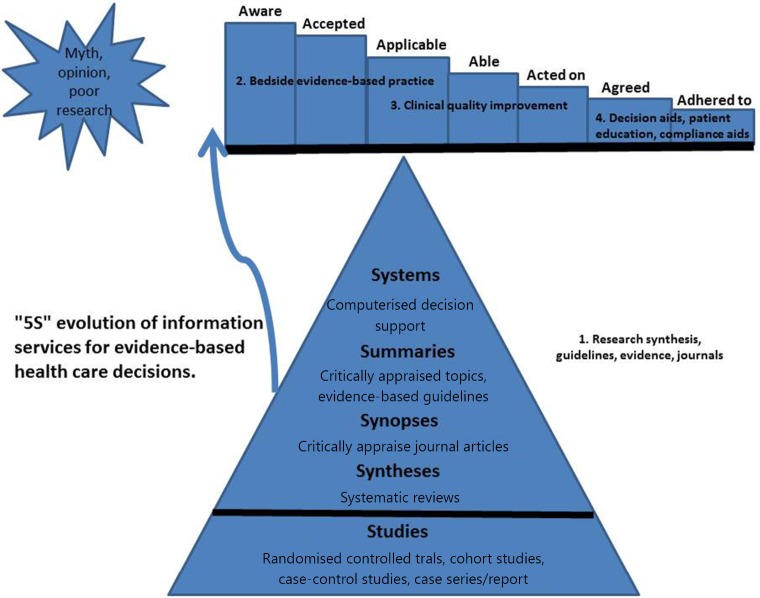
The ‘leaks’ between research and practice.

One of the key components of the EBM process is the application of appraisal results to clinical practice^6^. If we cannot achieve this outcome, even the most methodologically sound study becomes irrelevant and resources expended in data collection are wasted. To traverse the transition between evidence appraisal and changes in clinical practice, clinicians must perform several steps, which have been conceptualized in an acceptance-to-adherence model. In this model, the path from evidence to clinical action is considered an “evidence pipeline”, which may leak at any stage, losing the potential clinical benefits of empirical findings^59^^,^^60^ (**Figure 1**).

A clear example of such a leak is the evidence surrounding the use of anticholinergic medications for the treatment of excessive respiratory secretions or “the death rattle” at the end of life. A Cochrane systematic review of four randomized prospective intervention studies concluded that there was no evidence for the benefit of any pharmacological intervention for the treatment of noisy breathing at the end of life^61^. The majority of included studies were small, and only one study included more than 300 participants. Furthermore, only one study used a placebo control. However, the authors concluded that the likelihood of bias in the studies included was low, and the evidence that anti-muscarinic drugs have no proven benefit in treating the death rattle can be considered to be of reasonably high quality.

Despite this evidence, which was an update of a prior review with the same conclusion, the authors noted that the treatment is “*undertaken by palliative care physicians and nurses all over the world*”. As such, a discrepancy exists between the available best evidence and clinical practice. Whilst the paradigm of the Cochrane review is extremely useful for determining whether an intervention is beneficial, it is clearly unable to determine why in the face of such evidence practice remains unchanged. For this type of research question, qualitative research methods may be more suitable. Focus group discussions with staff members in inpatient palliative care services revealed that staff are largely negatively affected by the sound of excess respiratory secretions and believe that other patients and relatives will also be distressed by the sound^62^. Furthermore, in two separate focus group sessions, staff acknowledged that they felt pressured to administer or prescribe anticholinergic medications because of perceived pressure from the patient’s family, or because of a general feeling that it was necessary to “do something”, even if they had little belief in the effectiveness of the drug for the patient, or more generally^62^^,^^63^. Despite the lack of methodological rigor, this type of thematic analysis is extremely effective, when combined with empirical studies, at unpicking some of the subtle beliefs and values that contribute to the time-lag between research discoveries and a change in clinical behavior.

## EBM model is changing

At its best, EBM “provides a common language through which we can communicate”^6^. However, some scholars have increasingly suggested that the EBM model has become too focused on a “top-down” approach, which emphasizes “populations, statistics, risk, and spurious certainty”^64^, and has drifted away from its founding principles, in which high-level research is applied flexibly to the individual patient. The challenges of conducting research in palliative care presented in this review can be regarded not as examples of shortcomings of the field itself, but as exemplars of the rigidity of the principles that guide our attempts to obtain information and improve patient care. Greenhalgh^64^ calls for a return to the original principles of EBM, and suggests that “logic of care” must underpin all efforts to negotiate the collection and analysis of evidence, in which the goal is “good illness management”. In no area of medicine does this aim seem such appropriate as that of palliative care. How might we elucidate such “logic of care”? The Recent Medical Research Council guidelines^65^ address this with the recommendation that mixed methods incorporating qualitative and quantitative analyses must be used to assess all complex interventions. This approach has been adopted in the palliative care setting to investigate a complex intervention for breathlessness^66^^-^^68^, and provides evidence for the benefit of placing value on the whole range of research methodology. This set of studies and the aforementioned work by Wee *et al*.^61^^,^^62^^,^^69^ and Hirsch^63^ on respiratory secretions demonstrate the importance of establishing the underlying qualitative themes to direct quantitative research questions toward a deeper and more useful understanding of the complex landscape of palliative care.

## Conclusion

EBM has been increasingly accepted as the model for modern medical care. This approach is based on fundamental assumptions about the rigor of various research methodologies and relies upon the broad availability of literature that meets such pre-defined criteria of quality. It is becoming increasingly unacceptable to make clinical decisions in the absence of such “rules of evidence by which we can agree on who will do what to whom”^6^. However, clinicians in palliative care find themselves hampered in the age of EBM by a significant dearth of well-designed and adequately powered research on relevant and useful clinical questions. It remains true in 2015 that despite the growth in published literature, palliative care is not an evidence-based discipline, or at least it is not informed by the level of evidence which most would require to label it such. This is for entirely predictable and understandable reasons. The patient population, the unique aims of treatment and the trajectory of incurable disease all conspire to make the traditional “gold-standard” of RCTs a challenging and, at times, inappropriate model with which to drive improvements in care. Despite the challenges inherent in palliative care research, the increasingly collaborative and well-organized research community has recognized the need for high-quality research and has begun to produce examples of excellence. This has been achieved by a combination of resourcefulness, in which non-RCT methodologies of equivalent validity and size are utilized; collaboration, in which resources are pooled; and creativity, in which multiple research tools are used in combination with a “mixed method” approach^65^. The future of research in palliative care will include expansion of collaborative research networks, use of standardized data collection tools and registries, and development of an active EBM culture within palliative care organizations. The EBM model is also likely to grow more flexible as the traditional unimodal top-down approach is replaced by the use of multiple diverse methodologies as a research tool kit. Such a mixed-method approach will be viewed not as a means by which to dodge the challenge of conducting methodologically rigorous work in palliative care. Instead, a multi-faceted approach can be regarded as a more valuable scheme with which to address the complex and subtle questions that define the objective of the palliative care research agenda.
